# Why mental disorders are brain disorders. And why they are not: ADHD and the challenges of heterogeneity and reification

**DOI:** 10.3389/fpsyt.2022.943049

**Published:** 2022-08-22

**Authors:** Stephan Schleim

**Affiliations:** Theory and History of Psychology, Faculty of Behavioral and Social Sciences, Heymans Institute for Psychological Research, University of Groningen, Groningen, Netherlands

**Keywords:** ADHD, biologism, reductionism, reification, heterogeneity, mental disorders, essentialism, pragmatism

## Abstract

Scientific attempts to identify biomarkers to reliably diagnose mental disorders have thus far been unsuccessful. This has inspired the Research Domain Criteria (RDoC) approach which decomposes mental disorders into behavioral, emotional, and cognitive domains. This perspective article argues that the search for biomarkers in psychiatry presupposes that the present mental health categories reflect certain (neuro-) biological features, that is, that these categories are *reified* as biological states or processes. I present two arguments to show that this assumption is very unlikely: First, the *heterogeneity* (both within and between subjects) of mental disorders is grossly underestimated, which is particularly salient for an example like Attention Deficit/Hyperactivity Disorder (ADHD). Second, even the search for the biological basis of *psychologically more basic categories* (cognitive and emotional processes) than the symptom descriptions commonly used in mental disorder classifications has thus far been inconclusive. While philosophers have discussed this as the problem of mind-body-reductionism for ages, Turkheimer presented a theoretical framework comparing weak and strong biologism which is more useful for empirical research. This perspective article concludes that mental disorders are brain disorders in the sense of *weak*, but not *strong* biologism. This has important implications for psychiatric research: The search for reliable biomarkers for mental disorder categories we know is unlikely to ever be successful. This implies that biology is not the suitable taxonomic basis for psychiatry, but also psychology at large.

## Introduction

Attention Deficit/Hyperactivity Disorder (ADHD) is a mental disorder category introduced into the third edition of the Diagnostics and Statistical Manual of Mental Disorders (DSM), published in 1980 (1)^1^. This classification replaced earlier categories such as Hyperkinetic Disorder, Minimal Brain Dysfunction, or Minimal Brain Damage. Even further back in history, similar medical categories emphasized children's moral misbehavior more than their lack of attention (2). The present DSM-5-TR still lists ADHD as a *neurodevelopmental* disorder which was originally only diagnosed in children and adolescents (3), but the diagnosis now has become increasingly common in adults (4). While some researchers state to have finally shown that ADHD is a disorder of the brain (5, 6), others emphasize that there are no reliable biomarkers for the diagnosis of *any* mental disorder as classified in the DSM (7–11).

One of the major aims for the DSM-5 (12) was the introduction of a *psychiatric pathophysiology* to replace or at least complement clinical observation (13, 14). This article argues that a physiology- or biology-based taxonomy of diseases was and still is unlikely to emerge for mental disorders. The two central arguments, heterogeneity and conceptual irreducibility, will be illustrated for the example of ADHD, but can be extended to all mental disorders and even psychological processes at large. Note that these arguments are essentially conceptual and, in this sense, philosophical, but supported by empirical research. To illustrate this, the theoretical concept of *biologism* is introduced in the next section. This is then applied to ADHD and later backed up by research in cognitive neuroscience, before the article concludes with a summary and outlook.

## Weak and strong biologism

In a classic paper on heritability and biological explanation, Turkheimer coined the concepts of weak and strong biologism (15, 16). He argued from the position that somehow *all* of our behavior and psychology is heritable and biological: the correlation between variations in the *phenotype* (here: behaviors, psychological processes, symptoms, mental disorders) and variation in the *genotype* is never zero. If everything is “somehow heritable,” then the still commonly reported heritability estimates are less informative—and they often are even misunderstood (17). This view is compatible with the recently increasing trend to view humans and cognition as *embodied* (18–23) or, to apply the notion recently used by Hyman, *grounded* in biology (10). This roughly characterizes Turkheimer's weak biologism. By contrast, a much stronger link between biology and psychology is required in cases of strong biologism, such that a behavior or psychological process “is well-represented as a process at a biological level of explanation,” for example, by “identification of a structurally or functionally localized biological process that explains some large part of the high-level phenomenon” (16). To illustrate this, Turkheimer gives the example of a 70-year-old man who suffers from aphasia after a stroke in Broca's area. In this case the patient's language difficulties are explained for a large part by the neurological damage in his language-producing brain area.

Similar examples for strong biologism would be a certain genotype, a certain brain function or structure strongly correlated with a particular psychological process or behavior. Already many years ago, Kendler argued that it is unlikely to find genes for a mental disorder in this strong sense, because the effect sizes are way too small and genes are too causally remote from the phenomenon to be explained (24), which is now corroborated by analyses involving hundreds of thousands of people (25). The statistical correlations between genotype and phenotype for mental disorders are many, in some cases distributed over hundreds of genetic loci, but they are way too small to explain the “high-level phenomenon,” that is, the mental disorder. But note that the idea to base a mental disorder taxonomy on biology, to develop a psychiatric pathophysiology, is much older than the plans to compile the DSM-5 (13, 14); it is also older than the DSM-III which was motivated by the idea to develop, for the first time, a “science-based” psychiatric diagnostic manual (26); it is even older than Emil Kraepelin's (1856–1926) ground-breaking neurological research, which is often given as a historical reference for the search for biomarkers in psychiatry. Actually Wilhelm Griesinger (1817–1868), sometimes called the “father of neuropsychiatry” (27, 28), already wrote in 1845:

“The first step toward a knowledge of the symptoms is their locality—to which organ do the indications of the disease belong? what organ must necessarily and invariably be diseased where there is madness? The answer to these questions is preliminary to all advancement in the study of mental disease. Physiological and pathological facts show us that this organ can only be the brain; we therefore primarily, and in every case of mental disease, recognize a morbid action of that organ.” (29).

But Griesinger also conceded that “[a] classification of mental diseases according to their nature—that is, according to the anatomical changes of the brain which lie at their foundation—is, at the present time, impossible” (29). We now, 177 years later, know that this statement is still correct. Yet it is interesting to see how the idea of a psychiatric pathophysiology and in particular of the localizability of mental disorders in the brain spans such a long period of psychiatric history and is also reflected in Turkheimer's more recent description of strong biologism. Weak and strong biologism are theoretical concepts; that there are no reliable diagnostic biomarkers for mental disorders is an empirical fact. In the next section we will discuss the example of ADHD in more detail to make a first step toward developing a general argument.

## Biologism and ADHD

It has become popular to ask “what kind of things” mental disorders are (30). These authors commonly relate the question to the three general theoretical views of *essentialism, social constructionism*, and *pragmatism*, besides their own model that might be understood as an updated an integrative version of the *biopsychosocial model* (31, 32). Essentialism, briefly summarized, resembles the idea of there being reliable biomarkers for mental disorders, which allow to build a psychiatric taxonomy and at the same time are targets for treatment; for example, *broken neural circuits* in the brain would explain mental disorders in the sense of strong biologism (33, 34). Social constructionism in a strong form holds that mental disorders are what certain social institutions *define* them to be, with the pathologization of homosexuality being a prime example (35, 36). Pragmatism tries to avoid philosophical conundrums and focuses on what is useful for clinicians and/or patients. Many authors now seem to agree that essentialism has been an influential model to drive scientific research in the past decades, but yielded few clinically useful results, while constructionism and pragmatism are also insufficient, particularly regarding the development of a better taxonomy (10, 30, 37). Let us now discuss what kind of “thing” ADHD may be.

The presently used classification distinguishes two major types and a third mixed type of the disorder (3). The major types can be characterized as an 1) inattentive and 2) hyperactive/impulsive type, while the mixed type is 3) a combination of the former two. The two major types are characterized by nine symptoms each, of which at least six should be present as a precondition for the diagnosis. For example, 1) entails difficulty sustaining attention, not listening when spoken to directly, and often losing things necessary for tasks and activities; 2) entails leaving seat in situations when remaining seated is expected, often being unable to play quietly, and talking excessively. This list of 18 symptoms with the additional condition allows us to get a better understanding of the *heterogeneity* of the thus classified disorder. Similar to Major Depressive Disorder, of which there are 227 variants (38), we can distinguish 130 pure forms of ADHD for each major type. Combining each pure type of 1) with each pure type of 2) already adds 16,900 additional mixed types; including the remaining symptom combinations yields a total of 116,220^2^. In addition to this, the DSM lists “other specified” and “unspecified” forms of ADHD with even broader diagnostic criteria. Further dimensions of complexity and thus also heterogeneity are added by variations in *frequency* of the presence of symptoms as well as their *severity* (39). For example, children might be so inattentive that they regularly miss important details of classes at school—-or they might always miss all details. Finally, the problem of *comorbidity* also adds to this: It is well known that more than one disorder is often diagnosed at the same, in spite of the already hundreds of available categories in the DSM (40).

The complexity and heterogeneity described here must obviously be reflected in subjects' physiological processes, as required by soft biologism and embodiment alike. While these thoughts are insufficient to develop a strong logical proof, they make it unlikely to reduce the heterogeneity of a category like ADHD to one or a few reliable biomarkers. And while it is true that also classical somatic diseases such as influenza can be expressed in different individuals or even the same person differently, these cases are unified by the *common cause*, that is, the infection with an influenza virus. However, since the DSM-III it has been acknowledged that the causes of mental disorders are unknown (26) and we can only speak of causal or risk factors, which increase someone's likelihood of having certain symptoms. This section considered the heterogeneity related to *symptom combinations* subsumed under the classification of ADHD. The next section will go deeper and discuss neuroimaging results regarding *individual symptoms*.

## Biologism and cognitive neuroscience

Imagine a psychology student seeing a psychiatrist. After a clinical interview, the latter diagnoses ADHD of the inattentive type. The student, just coming from a journal club discussing Anderson's paper (41, 42), replies: “But doctor, there is no such thing as attention!” What could the psychiatrist say?

In this fictive but not entirely unrealistic example two different worlds clash: that of basic science trying to understand “how the mind works” and that of a clinician responding to a patient's immediate need. In the latter world, waiting until the foundational debates of the former world are solved is not an option. The example emphasizes the fact that even more basic than the question what kind of things mental disorders are (30), the more general question about the basic level of description for psychology altogether can be raised (8, 43–45). Skepticism about the scientific value of “mental vocabulary” has given rise to the influential behavioristic school of psychology some 100 years ago (46, 47). Interestingly, neuroscientists very recently voiced a similar critique of psychological vocabulary and reemphasized the importance of focusing on behavior (48, 49). Ever since psychologists used physiological measurements to better understand psychological processes, the problem persists that there is no one-to-one correspondence between psychological and physiological processes (50, 51). Thus, the quest for a psychiatric pathophysiology with reliable biomarkers is closely entangled with the quest for a biological taxonomy for psychology. Influenced by clinical observations after brain damage and the rise of modern brain scanning such as fMRI (52) hopes were high to classify psychological processes—and accordingly mental disorders—on the basis of brain processes. After some 30 years of fMRI and even more research with other techniques it once more becomes clear that there is no one-to-one correspondence to classify cognitive and emotional processes on the neural level (51, 53–55), though some researchers now pursue the hypothesis that more specificity can be found with *network analyses* (56, 57).

**Figure 1:**
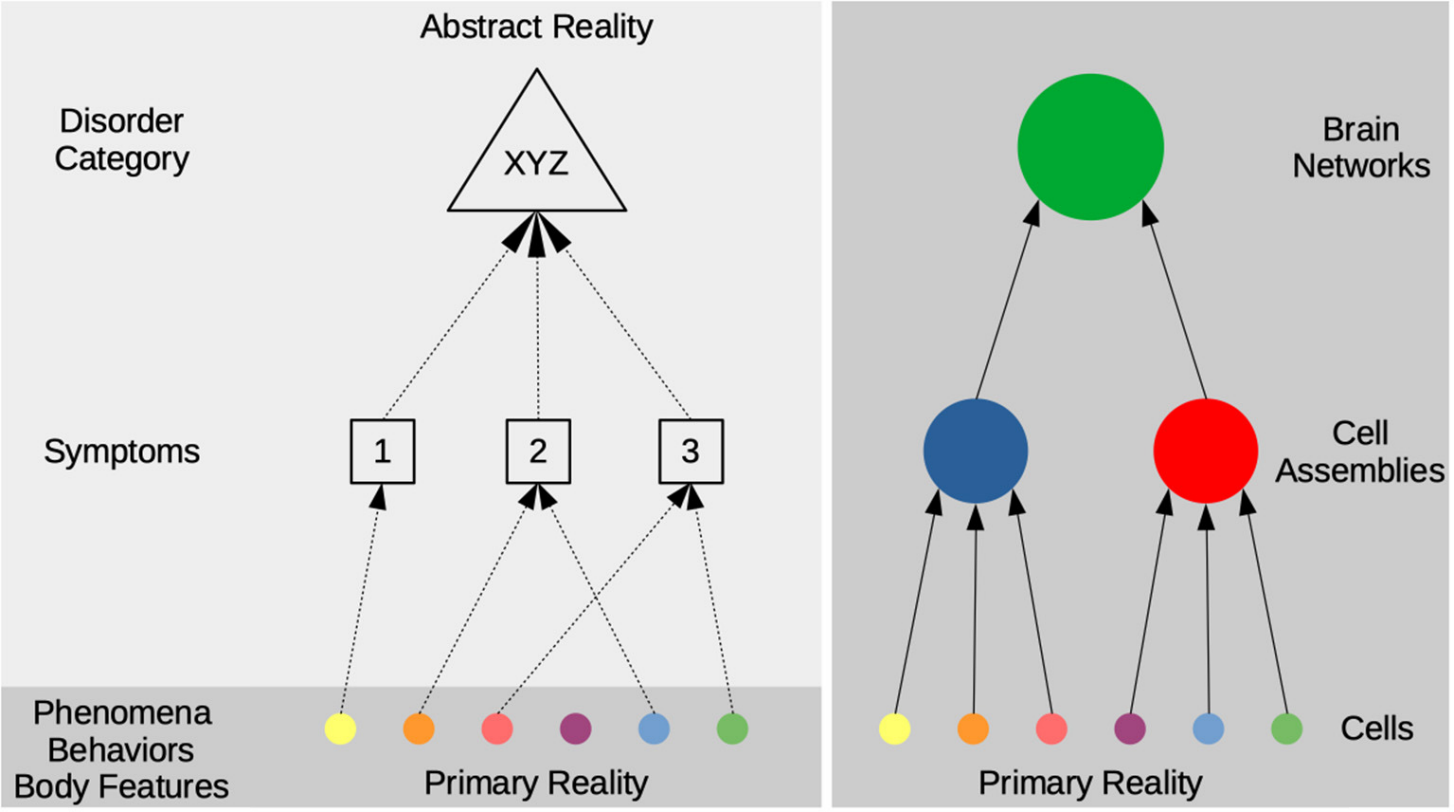
The scheme distinguishes a hierarchy from experiences (phenomena), behaviors, and body features to symptoms and disorder categories on the left side and a hierarchy in the brain from single cells to cell assemblies and whole brain networks on the right side. Note that the hierarchy on the left describes a relationship of *conceptual abstraction*, that is, symptom descriptions are intended to cover patients' *actual* states and processes and then combined into complex mental disorder classifications. By contrast, the hierarchy on the right describes a *part-whole relationship* (mereology) of actual things. The light and dark shaded gray backgrounds illustrate the difference between a more *primary* and more *abstract reality*: entities in the former are less dependent from our way of describing or classifying them as such. For the example of ADHD, phenomena/behaviors could be a girl's daydreaming or a boy's running around in class, which are subsumed under more general symptom descriptions by clinical experts and eventually codified as the disorder category. *Strong biologism* would require a strong correspondence between the entities on the left and the right side, but the disorders' heterogeneity and conceptual irreducibility make this very unlikely.

The previous section illustrated that mental disorder categories are such complex and heterogeneous entities that the discovery of reliable diagnostic biomarkers is unlikely, which is also supported by some 180 years of psychiatric history, though it should also be mentioned that diseases like epilepsy or Parkinson's which were originally understood as psychiatric disorders moved to neurology after the discovery of strong neural markers (58, 59). This section adds to that argument that the problem is not just one of complexity, but more generally one of our psychological language and its relation to the world (43, 60). The idea of a biological taxonomy for psychology and psychiatry then always carries the risk of prematurely *reifying* definitions of psychological processes or classifications of mental disorders, that is, the risk of treating them *as things* while they are in the first place pragmatic constructs to help scientists, clinicians, and patients fulfill their needs. Such a premature reification would also be at odds with, first, the history of changing definitions of mental disorders, as we have already seen for ADHD above, second, the introduction of new disorders and, third, the removal of others (61). Importantly, this does not make the psychological problems experienced by patients “only constructs,” although it is also well documented how culture and institutions interact with psychological problems (62–65)^3^. But it illustrates in a more general way that already symptoms as defined by clinicians—and even more so complex classifications of mental disorders—are entities of a different kind than people's behaviors and experiences on the one hand and neurons, neural networks, and brain areas on the other (Figure 1).

In line with weak biologism and embodiment, researchers are likely to always find *something* when they are looking for a biological correlate of psychological processes in general or mental disorders in particular. Note that this correlate (66), even though localized in the individual's body, might still be caused by external psychosocial factors such as serious life events or poverty (67, 68). Particularly for ADHD, children's age at school enrollment and poverty have been identified as strong predictors of a diagnosis (69, 70). But in contrast to the requirements of strong biologism, the then discovered biological something alone is insufficient to distinguish, explain, and, where necessary, treat a patient's problems. This conclusion is not only compatible with some 180 years of psychiatric research, but also the present situation in clinical neuroscience, particularly genetics and neuroimaging research (7, 8, 10, 25, 37). There are several possible ways for research to proceed in this situation, which will be described in the last section.

## Summary and outlook

This article started out with the finding that, in spite of intense efforts, reliable diagnostic biomarkers or a pathophysiology for mental disorders are still unavailable. This cannot only be explained by the complexity or heterogeneity of mental disorders, but even more generally by the relation between psychological language and bodies/brains. Attempts to find one-to-one or at least very specific many-to-one correspondence between brain and mind continue on the level of network analysis (49, 56). Yet to be discovered new neurobiological methods might also turn out to be a “game changer” in the future.

However, for the time being a decision needs to be taken about the priority of patients', clinicians', institutions', and scientists' needs: A more patient-based position should emphasize their problems and experience (phenomenology), what it is like for them to suffer from certain psychological problems, how to cope with, treat, and prevent them (71, 72). A continuation of the quest for biomarkers or “broken brain circuits” carries the risk of neglecting the patients' perspective and delaying clinical translation into an uncertain and far future (33, 34, 73). The biologization/medicalization of mental disorders has furthermore not solved the problem of stigmatization and can instead increase the social distance between patients and non-patients (74–77). The Research Domain Criteria (RDoC) comprise a more comprehensive, but still primarily neurobiological approach (78–80). The recently proposed focus on behavior^4^ might also neglect patients' experience and create situations in which someone gets a diagnosis without experiencing psychological problems, or experiencing problems without getting a diagnosis (38, 48, 49). A middle way with more balanced priorities could be the Hierarchical Taxonomy of Psychopathology (HiTOP) presently developed primarily by psychologists (81). Note that projects like RDoC and HiTOP imply, to a certain extent, to *change our language* such that it better fits some biological, psychological, and/or statistical categories.

This article explained that mental disorders such as ADHD are brain disorders in the sense of weak biologism and in line with embodiment: Our whole psychology is realized by a body, embedded in a complex environment, and in continuous interaction (16, 18–23, 71). It is important to stress that the decision which of these states to consider a *disorder* is not based on biological facts, but usually the social norms applied by clinical experts (82, 83). It should have become clear that mental disorders are not brain disorders in the sense of strong biologism, because of their heterogeneity and more generally the relationship between psychological language and biology. There is no guarantee that this conclusion will remain valid forever–but it is the one making the most sense in light of the historical and present evidence.

## Data availability statement

The original contributions presented in the study are included in the article/supplementary material, further inquiries can be directed to the corresponding author.

## Author contributions

SS conceived and wrote the whole manuscript.

## Funding

This publication has been supported by the History of Neuroethics grant by the Dutch Research Foundation (NWO), Grant Number 451-15-042.

## Conflict of interest

The author declares that the research was conducted in the absence of any commercial or financial relationships that could be construed as a potential conflict of interest.

## Publisher's note

All claims expressed in this article are solely those of the authors and do not necessarily represent those of their affiliated organizations, or those of the publisher, the editors and the reviewers. Any product that may be evaluated in this article, or claim that may be made by its manufacturer, is not guaranteed or endorsed by the publisher.

## Footnotes

^1^Strictly speaking, the DSM-III of 1980 distinguished ADD with or without hyperactivity. The text-revision of 1987 (DSM-III-R) then changed this into ADHD.

^2^The equation is: 2^*^130^*^2^9^-130^2^. Each of the 2^*^130 pure types is combined with any combination of the nine symptoms of the other type, which can be represented as a nine-digit binary number, thus 2^9^ possibilities; from this the redundant combinations must be subtracted.

^3^This “only constructs” response is also related to the sorrows that psychological or psychiatric phenomena might be considered as somehow “less real” without essentialism or something near enough (e.g. Kendler, 2016). Alternatively, one might consider to educate people that consciousness, subjectivity, and psychological processes are by no means less real. Instead, the point could be made that these phenomena are actually *more directly given* to us than knowledge of the “objective” world.

^4^An idea I cannot pursue in more detail here is that mental disorder classifications differ with respect to entailing symptoms about the body (e.g. weight gain or loss associated for MDD), psychological processes (e.g. attention for ADHD or depressive mood for MDD), or behaviors (e.g. inability to stay seated or impulsivity for ADHD). At first glance, behavioral features seem to be more salient in the classification of ADHD, particularly the hyperactive type.
